# Thermally-assisted photosensitized emission in a trivalent terbium complex

**DOI:** 10.1038/s42004-023-00922-5

**Published:** 2023-06-22

**Authors:** Yuichi Kitagawa, Kaori Shima, Takuma Nakai, Marina Kumagai, Shun Omagari, Pedro Paulo Ferreira da Rosa, Sunao Shoji, Koji Fushimi, Yasuchika Hasegawa

**Affiliations:** 1 Faculty of Engineering, Hokkaido University, N13W8, Kita-ku, Sapporo, Hokkaido 060–8628 Japan; 2 Institute for Chemical Reaction Design and Discovery (WPI-ICReDD), Hokkaido University, N21W10, Sapporo, Hokkaido 001-0021 Japan; 3 Graduate School of Chemical Sciences and Engineering, Hokkaido University, N13W8, Sapporo, Hokkaido 060-8628 Japan; 4 Department of Materials Science and Engineering, Tokyo Institute of Technology, Ookayama 2-12-1-S8-44, Meguro-ku, Tokyo, 152-8552 Japan; 5 Department of Engineering, Nara Women’s University, Kitauoya Nishimachi, Nara, 630-8506 Japan

**Keywords:** Light harvesting, Fluorescence spectroscopy, Energy transfer

## Abstract

Luminescent lanthanide complexes containing effective photosensitizers are promising materials for use in displays and sensors. The photosensitizer design strategy has been studied for developing the lanthanide-based luminophores. Herein, we demonstrate a photosensitizer design using dinuclear luminescent lanthanide complex, which exhibits thermally-assisted photosensitized emission. The lanthanide complex comprised Tb(III) ions, six tetramethylheptanedionates, and phosphine oxide bridge containing a phenanthrene frameworks. The phenanthrene ligand and Tb(III) ions are the energy donor (photosensitizer) and acceptor (emission center) parts, respectively. The energy-donating level of the ligand (lowest excited triplet (T_1_) level = 19,850 cm^−1^) is lower than the emitting level of the Tb(III) ion (^5^D_4_ level = 20,500 cm^−1^). The long-lived T_1_ state of the energy-donating ligands promoted an efficient thermally-assisted photosensitized emission of the Tb(III) acceptor (^5^D_4_ level), resulting in a pure-green colored emission with a high photosensitized emission quantum yield (73%).

## Abstract (web summary)

Luminescent lanthanide complexes are promising materials for use in displays and sensors, however, these materials often undergo thermal quenching. Here, the authors synthesize and characterize a terbium dinuclear phosphine oxide-bridged phenanthrene complex, which exhibits thermally-enhanced emission.

## Introduction

Highly luminescent molecules have become increasingly important for the development of display and sensing devices^1–6^. Numerous studies on luminescent molecular materials based on organic compounds and metal complexes have been reported^7–10^. Among these materials, visible luminescent lanthanide (where Ln(III) = Tb(III) and Eu(III)) complexes are considered promising candidates for highly luminescent molecules with high color purity originating from the intra-4f-orbital transitions^11–13^. However, they exhibit a small absorption coefficient (ε = 0.1–10 M^−1^ cm^−1^), which is mitigated by photosensitized energy transfer from organic ligands with a larger absorption coefficient (ε = 10^3^–10^5^ M^−1^ cm^−1^). Therefore, effective photosensitizer design is crucial for realizing strong lanthanide emissions.

**Fig. 1:**
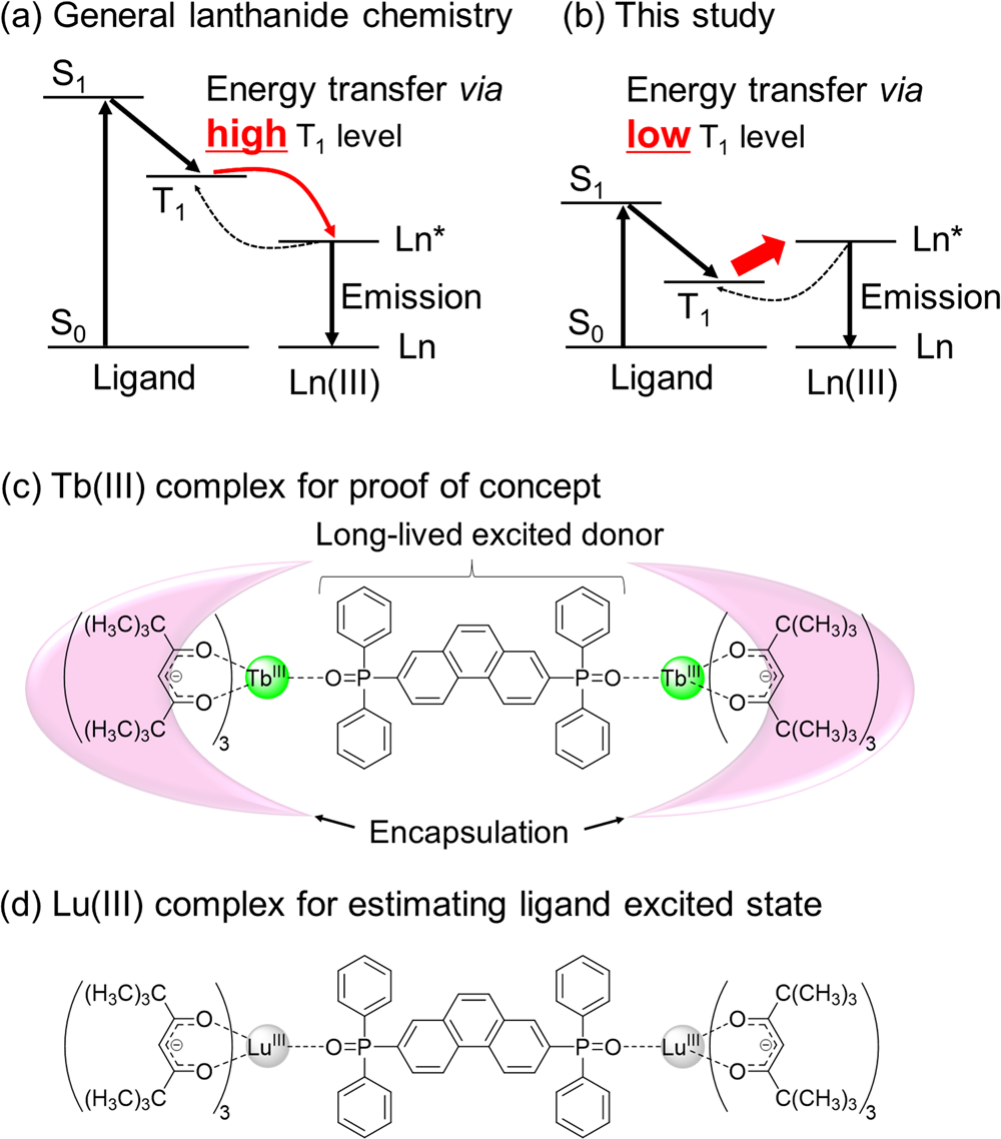
Photosensitizer design strategies. Schematic photosensitized emission mechanism based on energy transfer from high T_1_ level (**a**, general lanthanide chemistry) and low T_1_ level (**b**, this study). The chemical structures of Tb(III) complex for proof of concept (**c**) and Lu(III) complex for estimating ligand-excited states (**d**).

The organic ligands undergo intersystem crossing (ISC) from the lowest singlet excited state (S_1_) to the lowest triplet excited state (T_1_) after excitation, thereby transferring their electronic energy to the Ln(III) ion. Latva et al. conducted a detailed investigation of the relationship between the photosensitized emission efficiency and T_1_ level using green luminescent Tb(III) and amino-carboxylate-typed ligands^14^. They suggested that the energy of the T_1_ level should be enough higher than that of the emitting level of Tb(III) ions (^5^D_4_: 20,500 cm^−1^, Supplementary Note 1 and Fig. S1) for strong Ln(III) emission (Fig. 1a; required energy gap between donor and acceptor in case of Tb(III) complexes >1850 cm^−1^ (Latva’s empirical rule)). This photosensitized energy transfer system, requiring a high T_1_ level, causes a strong restriction of the organic ligand designs in lanthanide complexes^15–18^.

Herein, we focused on the long-lived excited organic donor system to break this photosensitizer design rule of luminescent Ln(III) complexes. Theoretical calculations have suggested that the T_1_-Ln(III) energy transfer rate is much higher than the inverse of the lifetime of the excited states of Ln(III) ions^19^. The long T_1_ lifetime should allow the efficient use of Ln(III) emitting photons, even in the case of a low T_1_ level, when an excited equilibration between T_1_ and Ln(III) emitting states is formed^20–26^. In this study, we demonstrated the photosensitized emission of the Tb(III) complex with a T_1_ level of an organic ligand lower than the Tb(III) emitting level for the first time, using the long-lived excited organic ligands (Fig. 1b and Supplementary Note 2).

To demonstrate our conceptual strategy, we designed the seven-coordinated Tb(III) complexes with a 2,2,6,6-tetramethyl-3,5-heptanedionate (tmh) and bidentate phosphine oxide-containing phenanthrene framework (dpph, Fig. 1c). A density functional theory calculation indicated that the T_1_ level of dpph is lower than that of a Tb(III) ion. The phosphine oxide-containing polyaromatic hydrocarbon framework also provides long-lived localized T_1_ states in lanthanide complexes, which function as effective energy donors^20,27,28^. Two-sided tmh ligands encapsulate the dpph ligand, thereby extending dpph’s T_1_ lifetime^29^. The Lu(III) complex with a closed 4f-electronic configuration was prepared to estimate dpph’s energy level and excited lifetime in an Ln(III) complex (Fig. 1d)^30^. The photosensitization mechanism presents new frontiers in the fields of molecular lanthanide photophysics and photofunctional material science.

## Results and discussion

### Coordination structure

**Fig. 2:**
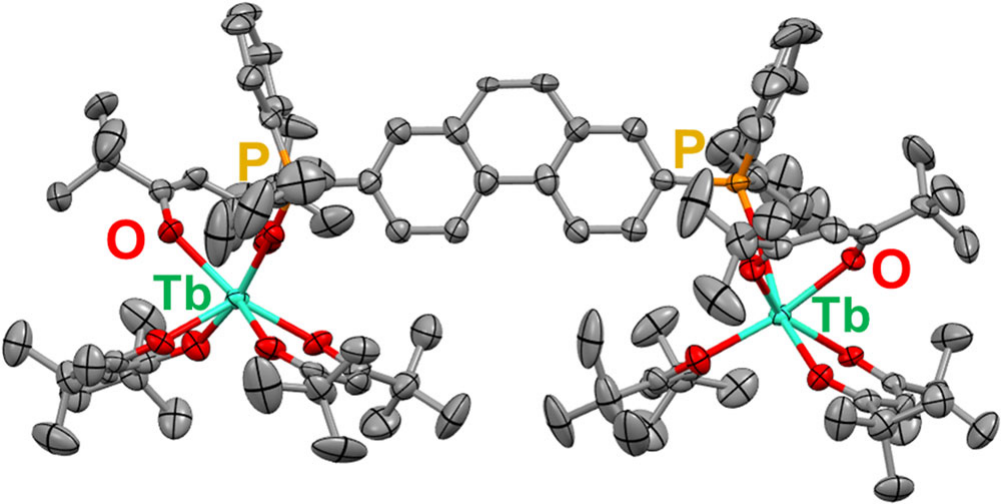
Crystal structure. ORTEP drawings (ellipsoids set at 50% probability) of **Tb-dpph** without hydrogen atoms. Gray spheres represent carbon; red spheres, oxygen; orange spheres, phosphorus; light green spheres, terbium.

The Tb(III)–Tb(III) and Lu(III)–Lu(III) dinuclear complexes were prepared by the complexation of [Tb_2_(tmh)_6_] and [Lu(tmh)_3_] with dpph in methanol, respectively (where [Tb_2_(tmh)_6_(dpph)]: **Tb-dpph**, and [Lu_2_(tmh)_6_(dpph)]: **Lu-dpph**). Single crystals of the dinuclear Tb(III) complex were obtained by recrystallization from the methanol solution. The crystal structure of **Tb-dpph**, shown in Fig. 2, was found to be triclinic, with the space group being P-1 (for the crystallographic data, see Table S1, ESI†). The coordination site in the Tb(III) complex comprised three tmh ligands and one phosphine oxide ligand. The single-crystal structure of the as-obtained **Lu-dpph** is almost the same as that of **Tb-dpph** (Fig. S2).

### Photophysical properties of ligand-excited states

**Fig. 3:**
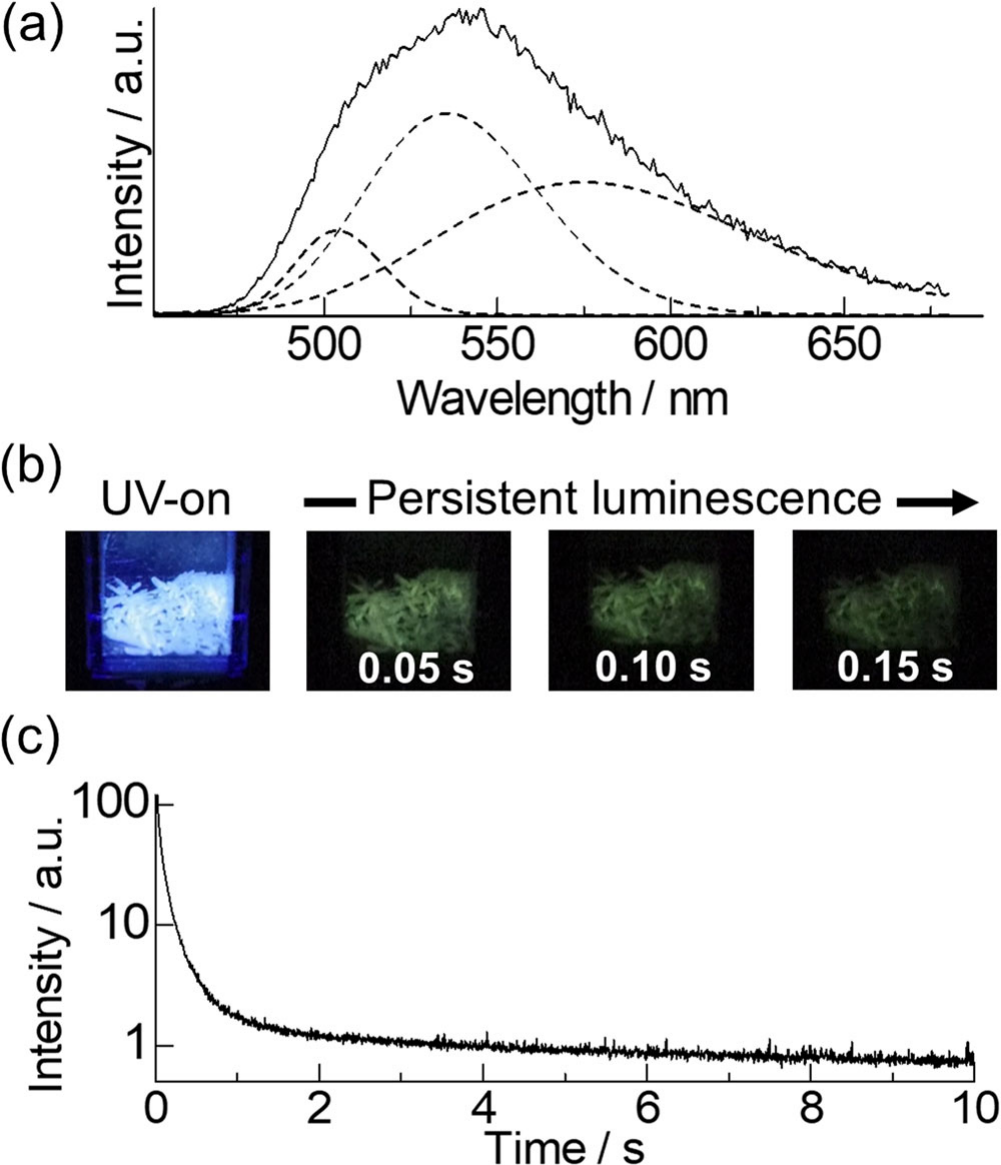
Photophysical properties of a ligand localized excited state. The emission spectrum of **Lu-dpph** (**a**: λ_ex_ = 400 nm; delay: 80 ms; 100 K). Emission images of **Lu-dpph** excited using UV-light (**b**: λ_ex_ = 375 nm; 293 K) under vacuum conditions. Emission-decay curves of **Lu-dpph** (**c**: λ_ex_ = 400 nm; λ_em_ = 530 nm; 293 K).

The emission spectrum of **Lu-dpph**, in degassed condition, is shown in Fig. 3a (solid line). The **Lu-dpph** shows a broad band at around 550 nm, which originated from π-π* transition of dpph ligand moiety (Supplementary Note 3 and Fig. S3–S5). The emission spectrum was deconvoluted into three vibronic bands using the software (OriginPro 2021b), the spectrum in wavenumber scale, and by fitting the peak profile using Gaussian functions (Fig. 3a, broken line). The deconvolution results in the three vibronic bands were designated as 0–0 (19,850 cm^−1^), 0–1 (18,670 cm^−1^), and 0–2 (17,390 cm^−1^). Thus, the T_1_ level of the dpph ligand in **Lu-dpph** was determined to be 19,850 cm^−1^ using band-deconvolution analysis. The emission photograph of **Lu-dpph** is shown in Fig. 3b, where it shows a green persistent luminescence.

The emission durability of **Lu-dpph** was evaluated using time-resolved emission spectroscopy (Fig. 3c), yielding characteristic emission-decay curves for persistent-emission materials (Supplementary Note 4 and Figs. S6–S8). Herein, the emission lifetimes were estimated using triple exponential functions (τ_1_ = 16 ms (70 %), τ_2_ = 83 ms (27%), and τ_3_ = 450 ms (3%)). The average π–π* emission lifetime of the dpph ligand in **Lu-dpph** was estimated to be 47 ms, which is characteristic among the T_1_ lifetime of organic ligands in lanthanide complexes at room temperature^30–33^. The long T_1_ lifetime in the dpph moiety was ascribed to the rigid isolated polyaromatic structure encapsulated in the tmh ligands, which suppressed the non-radiative deactivation pathways^29,34,35^. These results indicate the construction of an energy-donating system with a long T_1_ lifetime in **Tb-dpph**. This dpph T_1_ lifetime (47 ms) is significantly longer than the 4f–4f emission lifetimes of reported Tb(III) complexes^11,36^.

### Photophysical properties of a trivalent terbium complex

**Fig. 4:**
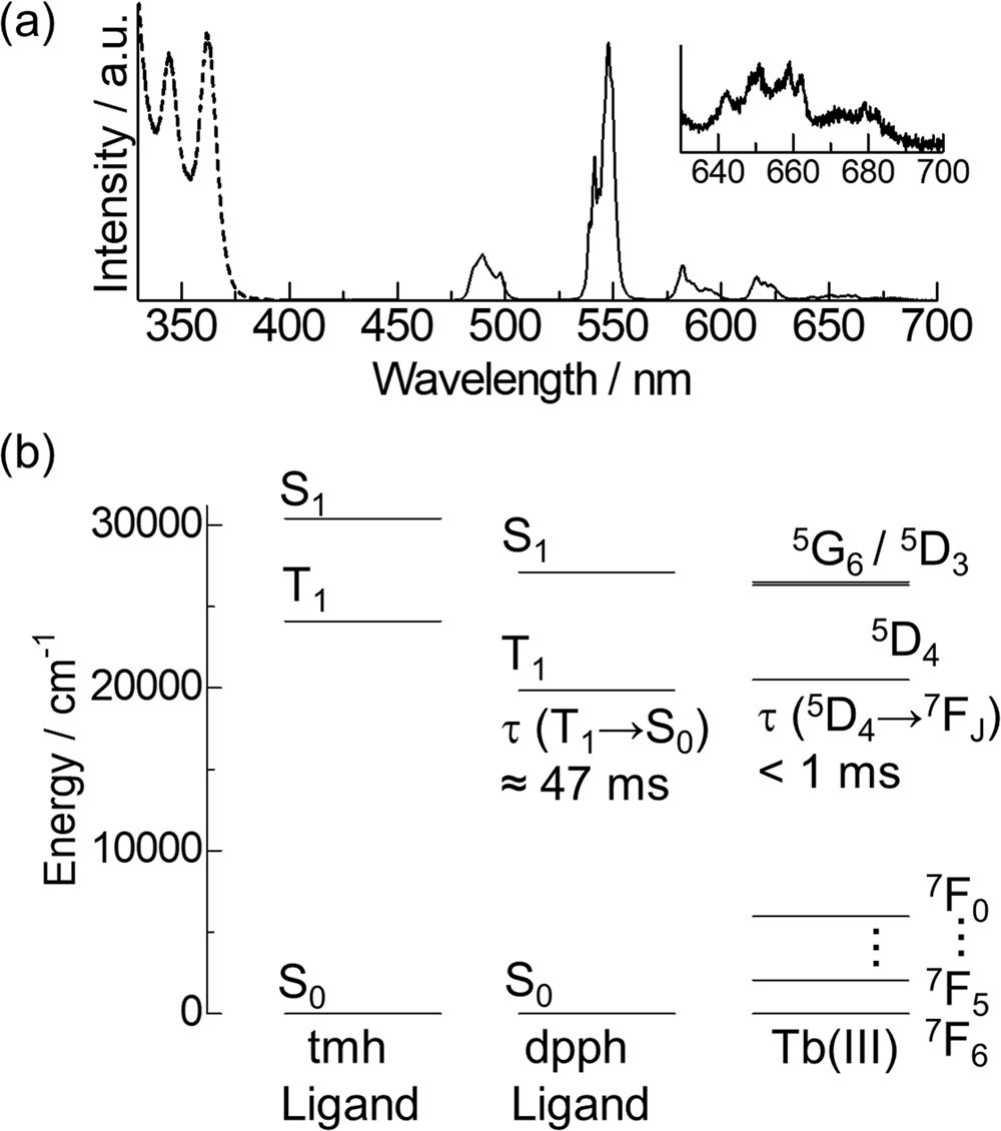
Photophysical properties of a trivalent terbium complex. Emission (solid line) and excitation (broken line) spectra of **Tb-dpph** (**a**: λ_ex_ = 356 nm; λ_em_ = 548 nm; 293 K). **b** The energy diagram for **Tb-dpph**.

The emission and excitation spectra of **Tb-dpph** in degassed conditions are shown in Fig. 4a. Sharp emission bands at 490, 548, 583, 616, 651, and 679 nm were observed for **Tb-dpph**, which are assigned to the ^5^D_4_ → ^7^F_6_, ^5^D_4_ → ^7^F_5_, ^5^D_4_ → ^7^F_4_, ^5^D_4_ → ^7^F_3_, ^5^D_4_ → ^7^F_2_, and ^5^D_4_ → ^7^F_1,0_ transitions of Tb(III), respectively. The observed excitation spectral bands at 344 and 362 nm are consistent with the absorption bands of the dpph ligand (Fig. S9), indicating energy transfer from the π-conjugated dpph ligand to Tb(III). The emission quantum yield and emission lifetime of **Tb-dpph** excited by the dpph ligand are estimated to be 73% and 0.83 ms, respectively. Thus, we successfully demonstrated a strong photosensitized emission using the energy-donating ligand with a lower T_1_ level than the emitting level of Tb(III).

### Mechanistic study

To understand this characteristic energy migration system, we evaluated the photophysical properties of the **Tb-dpph** excited by dpph ligand under the presence of oxygen. An excited state equilibrium between Tb(III) and ligand T_1_ was revealed through the emission lifetime measurements based on the oxygen concentrations^20,22–26^ (Fig. S10, Ar: 0.83 ms, Air: 0.57 ms). The photosensitized emission quantum yield was also dependent on the oxygen concentrations (Ar: 73%, Air: 57%). The energy diagram for **Tb-dpph** is shown in Fig. 4b. From the fluorescence measurements (Fig. S11), the S_1_ level of the dpph ligand (27,100 cm^−1^) is lower than that of the tmh ligand (30,400 cm^−1^). These results demonstrate that the effective photosensitized energy transfer occurs via the T_1_ state of the dpph moiety in the Tb(III) complex. The T_1_ level of the dpph ligand (19,850 cm^−1^) is much lower than that of the tmh ligand (24,400 cm^−1^)^37^, hence indicating that the energy transfer pathway from the dpph to the tmh ligand is negligible. To further understand the excited state dynamics, we evaluated the temperature dependence of the emission intensity and 4f–4f emission lifetimes (Supplementary Notes 5, 6 and Figs. S12–18). The photosensitized emission intensity increased with the temperature from 100 to 400 K, suggesting the existence of a thermally-enhanced photosensitization pathway such as intersystem crossing^38^ and/or energy transfer from T_1_. The temperature-dependent emission measurement by direct 4f–4f excitation revealed the existence of a thermally-enhanced emission via the T_1_ state in the excited-state equilibrium. The results suggest the existence of an endothermic energy transfer pathway corresponding to the ^7^F_6_ → ^5^D_4_ transitions (Supplementary Note 7, Fig. S19, and Table S3). However, time-resolved emission spectroscopy showed a temperature-insensitive emission lifetime (100–350 K) at the excited-state equilibrium with the long-lived excited state of the dpph ligand (Supplementary Note 6 and Figs. S16–18). The results suggest unusually efficient exothermic energy transfer pathways corresponding to the ^7^F_5_ → ^5^D_4_ transitions from the T_1_ states (+ΔE = 1400 cm^−1^) besides the endothermic energy transfer pathways corresponding to the ^7^F_6_ → ^5^D_4_ transitions from the T_1_ states (−ΔE = 650 cm^−1^). Theoretical studies suggest significantly populated ^7^F_5_ owing to the long decay lifetime of ^7^F_5_ → ^7^F_6_ in a relatively large energy gap between them (ca. 2050 cm^-1^)^39,40^, allowing energy transfer from ^7^F_5_ level^41^. Theoretical studies also indicate a larger energy-transfer matrix element for the ^7^F_5_ → ^5^D_4_ transition than that for the ^7^F_6_ → ^5^D_4_ transition^42^. The energy transfer from the ^7^F_5_ state is one of the models for explaining the present temperature-insensitive lifetime behavior (the detailed discussion in Supplementary Note 6). Considering the temperature-dependent photophysical measurements and theoretical aspects, the characteristic thermally-assisted photosensitized emission occurs via the dpph T_1_ state. Although determining the exact photosensitization pathway is difficult, this is, to the best of our knowledge, the first example of efficient photosensitized emission via the T_1_ state in a lanthanide complex with an organic ligand T_1_ level lower than the emitting level of the Ln(III) ion (Supplementary Note 8 and Figs. S20, S21).

## Conclusions

In this study, an effective photosensitized emission in a luminescent lanthanide complex with a T_1_ level of an organic ligand lower than the emitting level of an Ln(III) ion was demonstrated. The thermally-assisted photosensitized emission was based on the excited-state equilibrium between a luminescent Ln(III) ion and an organic ligand with a persistent excited state. The photosensitizer model with a low T_1_ level is advantageous for the construction of low-energy-driven photosensitization (Supplementary Note 9 and Fig. S22). The present study not only breaks the historical photosensitizer design rule based on Latva’s rule, but also presents a novel photosensitizer model for photofunctional materials beyond lanthanide photochemistry.

## Methods

### General methods

^1^H NMR spectrum was recorded in chloroform-d on a JEOL ECS-400 (400 MHz) spectrometer; TMS (δ_H_ = 0 ppm) was used as the internal standard. Electrospray ionization (ESI) mass spectrometry were performed using the JEOL JMS-T100 LP instrument. Elemental analyses were performed using MICRO CORDER JM10. Emission spectra (λ_ex_ = 356 nm), excitation spectra (λ_em_ = 548 nm), and emission lifetimes (λ_ex_ = 356 nm and λ_em_ = 548 nm) for [Tb_2_(tmh)_6_(dpph)] were measured using a Horiba FluoroLog®3 spectrofluorometer. Temperature-dependent emission spectra for [Tb_2_(tmh)_6_(dpph)] (λ_ex_ = 356 nm and λ_ex_ = 482 nm) were measured using a Horiba FluoroLog®3 spectrofluorometer with a cryostat (Thermal Block Company SA-SB245T) and a temperature controller (Scientific Instruments Model 9700). Temperature-dependent emission lifetimes for [Tb_2_(tmh)_6_(dpph)] were measured using the third harmonics (λ_ex_ = 355 nm) of a Q-switched Nd:YAG laser (Spectra Physics, INDI-50, fwhm = 5 ns, λ = 1064 nm) and a photomultiplier (Hamamatsu Photonics, R5108, response time ≤1.1 ns) with a cryostat (Thermal Block Company SA-SB245T) and a temperature controller (Scientific Instruments Model 9700). The Nd:YAG laser response was monitored with a digital oscilloscope (Sony Tektronix, TDS3052, *f* = 500 MHz) synchronized to the single-pulse excitation. Emission quantum yields for [Tb_2_(tmh)_6_(dpph)] (λ_ex_ = 370 nm) and [Lu_2_(tmh)_6_(dpph)] (λ_ex_ = 400 nm) were measured using FP-6300 spectrofluorometer with an integration sphere (ILF-533). Emission spectrum (λ_ex_ = 400 nm, 100 K, delay: 80 msec) and time-resolved emission intensity (λ_ex_ = 400 nm, λ_em_ = 530 nm, 293 K) for [Lu_2_(tmh)_6_(dpph)] were measured using FP-6300 spectrofluorometer with a cryostat (Thermal Block Company SA-SB245T) and a temperature controller (Scientific Instruments Model 9700). Emission lifetimes were estimated using triple exponential functions in the region from 0.02 to 10 s based on the time-delayed-dependent emission spectral results (Fig. S8). The percentage of emission intensity contribution (~0 s) was calculated using the estimated triple exponential function. Emission images of [Lu_2_(tmh)_6_(dpph)] were taken by a camera (PENTAX, K-70).

### Materials

Lutetium(III) nitrate hydrate (99.999%) was purchased from Aldrich Co., Ltd. Terbium(III) chloride hexahydrate (99.95%), *n*-butyllithium in *n*-hexane (1.6 mol/L), and chloroform-*d* (99.8%) were purchased from Kanto Chemical Co., Inc. Tetrahydrofuran, super dehydrated, with a stabilizer (for organic synthesis), and hydrogen peroxide (30%), sodium sulfate, anhydrous were purchased from Wako Pure Chemical Industries, Ltd. 2,2,6,6-Tetramethyl-3,5-heptanedione (>97%), 2,7-dibromophenanthrene (>98.0%) and chlorodiphenylphosphine (>97.0%) were purchased from Tokyo Chemical Industry Co., Ltd.

### Preparation of [2,7-bis(diphenylphosphoryl)phenanthrene (dpph)]

A solution of *n*-butyllithium (6.2 mL, 9.9 mmol) was added dropwise to a solution of 2,7-dibromophenanthrene (1.67 g, 4.97 mmol) in dry tetrahydrofuran (45 mL) at –76 °C under Ar atmosphere. After 2 h, chlorodiphenylphosphine (1.8 mL, 9.8 mmol) was added to the solution at –76 °C under Ar atmosphere, and then stirred for 20 h at room temperature. The reaction mixture was added to dichloromethane, washed with water, and then dried over anhydrous sodium sulfate. The obtained solution was evaporated and chloroform (30 ml) was added to the product. A 30% hydrogen peroxide aqueous solution (4 mL) was added to the solution, and the reaction mixture was stirred for 2 h. The product was extracted using dichloromethane, and the extract was washed with water and then dried over anhydrous sodium sulfate. The compounds were purified by silica gel column chromatography (ethyl acetate: methanol = 23: 2) (Yield: 63.8%, 1.83 g, 3.16 mmol).

^1^H NMR (400 MHz, chloroform-*d*) δ/ppm = 8.75 (dd, *J* = 8.8 Hz, 2.4 Hz, 2H), 8.31 (dd, *J* = 13.4 Hz, 1.0 Hz, 2H), 7.88 (t, *J* = 9 Hz, 2H), 7.79–7.47 (m, 22H); ESI-MS: *m*/*z* calcd. for [C_38_H_29_O_2_P_2_]^+^ = 579.16; found: 579.16. Elemental analysis calcd. (%) for C_38_H_28_O_2_P_2_, C 78.88, H 4.88; found: C 78.11, H 4.98.

### Preparation of [Tb_2_(tmh)_6_(dpph)]

Methanol solution (6 mL) containing Tb_2_(tmh)_6_ (99.2 mg, 0.07 mmol) and dpph (40.5 mg, 0.07 mmol) was refluxed for 18 h. The solution was filtered, and recrystallization from the solution gave white crystals (Yield: 41.1%, 57.5 mg, 0.0288 mmol).

ESI-MS: *m*/*z* calcd. for [C_82_H_104_O_10_P_2_Tb_2_]^2+^ = 814.28; found: 814.29. Elemental analysis calcd. (%) for C_104_H_142_Tb_2_O_14_P_2_, C 62.58, H 7.17; found: C 62.25, H 7.14; IR(ATR) = 2961 (st, C-H), 1575 (st, C=O), 1183 cm^−1^ (st, P=O).

### Preparation of [Lu_2_(tmh)_6_(dpph)]

Methanol solution (5 mL) containing Lu(tmh)_3_ (101.6 mg, 0.14 mmol) and dpph (40.6 mg, 0.07 mmol) was refluxed for 16 h. The solution was filtrated, and recrystallization from the solution gave white crystals (Yield: 14.1%, 20.1 mg, 0.0099 mmol).

ESI-MS: *m*/*z* calcd. for [C_82_H_104_Lu_2_O_10_P_2_]^2+^ = 830.29; found: 830.28, Elemental analysis calcd. (%) C_104_H_142_Lu_2_O_14_P_2_, C 61.59, H 7.06; found: C 61.19, H 7.06.

### Single-crystal X-ray structure determination

X-ray crystal structures for [Tb_2_(tmh)_6_(dpph)] and [Lu_2_(tmh)_6_(dpph)] are shown in Fig. 2 and Fig. S2, respectively. The crystallographic data are shown in Table S1. Single crystal X-ray diffraction data were obtained using Rigaku XtaLAB Synergy-DW equipped with a HyPix-6000HE detector (MoK_α_ radiation, λ = 0.71073 Å). Non-hydrogen atoms were refined anisotropically using the SHELX system. Hydrogen atoms were refined using the riding model. All calculations were performed using the crystal structure crystallographic and Olex 2 software package. The CIF data were confirmed by the check CIF/PLATON service.

## Supplementary information

Peer Review File

Supplementary Information

Description of additional supplementary files

Supplementary Data 1

Supplementary Data 2

## Data Availability

The single-crystal data generated in this study have been deposited in The Cambridge Crystallographic Data Center under accession code CCDC-2128731 (for [Tb_2_(tmh)_6_(dpph)], Supplementary Data 1) and CCDC-2128735 (for [Lu_2_(tmh)_6_(dpph)], Supplementary Data 2). These data can be obtained free of charge from The Cambridge Crystallographic Data Center via www.ccdc.cam.ac.uk/data_request/cif. All of the other data supporting the findings of this study are available from the corresponding author upon reasonable request.

## References

[1] Qi Y (2021). Recent advances in reaction-based fluorescent probes for the detection of central nervous system-related pathologies in vivo. Coord. Chem. Rev..

[2] Zhang YX, Qiao J (2021). Near-infrared emitting iridium complexes: Molecular design, photophysical properties, and related applications. iScience.

[3] Liu C, Yang J-C, Lam JWY, Feng H-T, Tang BZ (2022). Chiral assembly of organic luminogens with aggregation-induced emission. Chem. Sci..

[4] Gao C (2021). Application of triplet–triplet annihilation upconversion in organic optoelectronic devices: advances and perspectives. Adv. Mater..

[5] Nakanotani H, Tsuchiya Y, Adachi C (2021). Thermally-activated delayed fluorescence for light-emitting devices. Chem. Lett..

[6] Guo C, Sedwick AC, T H, Sessler JL (2021). Supramolecular fluorescent sensors: an historical overview and update. Coord. Chem. Rev..

[7] Uoyama H, Goushi K, Shizu K, Nomura H, Adachi C (2012). Highly efficient organic light-emitting diodes from delayed fluorescence. Nature.

[8] Kondo Y (2019). Narrowband deep-blue organic light-emitting diode featuring an organoboron-based emitter. Nat. Photon..

[9] Lustig WP (2017). Metal-organic frameworks: functional luminescent and photonic materials for sensing applications. Chem. Soc. Rev..

[10] Rocha J, Carlos LD, Paz FAA, Ananias D (2011). Luminescent multifunctional lanthanides-based metal-organic frameworks. Chem. Soc. Rev..

[11] Bünzli J-CG (2015). On the design of highly luminescent lanthanide complexes. Coord. Chem. Rev..

[12] Binnemans K (2015). Interpretation of europium(III) spectra. Coord. Chem. Rev..

[13] Moore EG, Samuel APS, Raymond KN (2009). From antenna to assay: lessons learned in lanthanide luminescence. Acc. Chem. Res..

[14] Latva M (1997). Correlation between the lowest triplet state energy level of the ligand and lanthanide(III) luminescence quantum yield. J. Lumin..

[15] Shavaleev NM, Eliseeva SV, Scopelliti R, Bünzli J-CG (2009). Designing simple tridentate ligands for highly luminescent europium complexes. Chem. Eur. J..

[16] Sato S, Masanobu W (1970). Relations between intramolecular energy transfer efficiencies and triplet state energies in rare earth β-diketone chelates. Bull. Chem. Soc. Jpn..

[17] Shavaleev NM, Eliseeva SV, Scopelliti R, Bünzli JCG (2010). *N*-Aryl chromophore ligands for bright europium luminescence. Inorg. Chem..

[18] Kitagawa M, Tsurui M, Hasegawa Y (2022). Bright red emission with high color purity from Eu(iii) complexes with π-conjugated polycyclic aromatic ligands and their sensing applications. RSC Adv..

[19] Malta OL (2018). Mechanisms of non-radiative energy transfer involving lanthanide ions revisited. J. Non-Cryst. Solids.

[20] Kitagawa Y (2020). Stacked nanocarbon photosensitizer for efficient blue light excited Eu(III) emission. Commun. Chem..

[21] Adachi C (2001). Endothermic energy transfer: a mechanism for generating very efficient high-energy phosphorescent emission in organic materials. Appl. Phys. Lett..

[22] Sørensen TJ, Kenwright AM, Faulkner S (2015). Bimetallic lanthanide complexes that display a ratiometric response to oxygen concentrations. Chem. Sci..

[23] Hueting R, Tropiano M, Faulkner S (2014). Exploring energy transfer between pyrene complexes and europium ions - potential routes to oxygen sensors. RSC Adv..

[24] Sabbatini N, Guardigli M, Manet I, Bolletta F, Ziessel R (1994). Synthesis and luminescence of lanthanide complexes of a branched macrocyclic ligand containing 2,2’-bipyridine and 9-methyl-1,10-phenanthroline subunits. Inorg. Chem..

[25] Lehr J, Tropiano M, Beer PD, Faulkner S, Davis JJ (2015). Ratiometric oxygen sensing using lanthanide luminescent emitting interfaces. Chem. Commun..

[26] Parker D, Fradgley JD, Wong K-L (2021). The design of responsive luminescent lanthanide probes and sensors. Chem. Soc. Rev..

[27] Kitagawa Y, Suzue F, Nakanishi T, Fushimi K, Hasegawa Y (2018). A highly luminescent Eu(III) complex based on an electronically isolated aromatic ring system with ultralong lifetime. Dalton Trans..

[28] Kitagawa Y, Kumagai M, Nakanishi T, Fushimi K, Hasegawa Y (2020). The role of π-f orbital interactions in Eu(III) complexes for an effective molecular luminescent thermometer. Inorg. Chem..

[29] Yang XG (2019). Facile synthesis of micro-scale MOF host-guest with long-last phosphorescence and enhanced optoelectronic performance. Chem. Commun..

[30] Kalota B, Tsvirko M (2015). Fluorescence and phosphorescence of lutetium(III) and gadolinium(III) porphyrins for the intraratiometric oxygen sensing. Chem. Phys. Lett..

[31] Sun B (2019). Highly efficient room-temperature phosphorescence achieved by gadolinium complexes. Dalton Trans..

[32] Zhao ZL (2019). A smart nanoprobe based on a gadolinium complex encapsulated by ZIF–8 with enhanced room temperature phosphorescence for synchronous oxygen sensing and photodynamic therapy. Dalton Trans..

[33] Kitagawa Y (2022). Effective photosensitization in excited‐state equilibrium: brilliant luminescence of Tb^III^ coordination polymers through ancillary ligand modifications. ChemPlusChem.

[34] Mieno H, Kabe R, Notsuka N, Allendorf MD, Adachi C (2016). Long-lived room-temperature phosphorescence of coronene in zeolitic imidazolate framework ZIF-8. Adv. Opt. Mater..

[35] Hirata S, Vacha M (2017). White Afterglow room-temperature emission from an isolated single aromatic unit under ambient condition. Adv. Opt. Mater..

[36] Yanagisawa K (2015). Seven-coordinate luminophores: brilliant luminescence of lanthanide complexes with C_*3v*_ geometrical structures. Eur. J. Inorg. Chem..

[37] Yanagisawa K (2017). Enhanced luminescence of asymmetrical seven-coordinate Eu^III^ complexes including LMCT perturbation. Eur. J. Inorg. Chem..

[38] Widman RP, Huber JR (1972). Temperature effects in the intersystem crossing process of anthracene. J. Phys. Chem..

[39] Rademaker K (2004). Optical properties of Nd^3+^ and Tb^3+^-doped KPb_2_Br_5_ and RbPb_2_Br_5_ with low nonradiative decay. J. Opt. Soc. Am. B.

[40] Roy UN (2005). Tb^3+^-doped KPb_2_Br_5_: low-energy phonon mid-infrared laser crystal. Appl. Phys. Lett..

[41] Carneiro Neto AN (2022). On the long decay time of the ^7^F_5_ level of Tb^3+^. J. Lumin..

[42] Moura RT (2021). Theoretical evidence of the singlet predominance in the intramolecular energy transfer in ruhemann’s purple Tb(III) complexes. Adv. Theory Simul..

