# Supplementary material for: Thermally-assisted photosensitized emission in a trivalent terbium complex
Source: Commun Chem. 2023 Jun 22;6:122. doi: 10.1038/s42004-023-00922-5 (PMC10287744; doi:10.1038/s42004-023-00922-5)
Supplement: Supplementary file 2 — Supplementary Information [file 42004_2023_922_MOESM2_ESM.pdf]

Supplementary Note 1. Energy levels of Ln(III) center (Ln(III) = Tb(III) and Lu(III))

The electronic configuration of trivalent lanthanide ions is $[Xe]4f^n$ (n = 0–14). The $4f^n$ configurations generate various electronic levels characterized by three quantum numbers, S, L, and J. For Tb(III) ($[Xe]4f^8$), the J levels of the ^7F and ^5D terms are described by the Russell–Saunders coupling scheme. The possible J values for the ^7F term are 0, 1, 2, 3, 4, 5, 6, so that the order of energies of the levels within the ^7F term is $^7F_6 < ^7F_5 < \cdots < ^7F_0$ based on the Hund's rule. Similarly, the lowest energy level of the ^5D term is $^5D_4$, which is corresponding to the emitting level of Tb(III). The Tb(III) emission bands are observed by the transition from the $^5D_4$ level to ^7F_J (J = 0, 1, 2, 3, 4, 5, 6) level.[S1-S2] The 4f-5d excited levels of Tb(III) (>30,000 cm^{-1}) are higher than the dpph ligand S_1 and 4f-4f excited states[S3], which do not affect the excited state dynamics directly. The energy diagram of the Tb(III) ion is shown in Figure S1. In contrast, Lu(III) with a closed 4f-electronic configuration does not have the 4f-4f excited state. The 4f-5d excited energy levels of Lu(III) (>80,000 cm^{-1}) is much higher than ligand excited state.[S3]

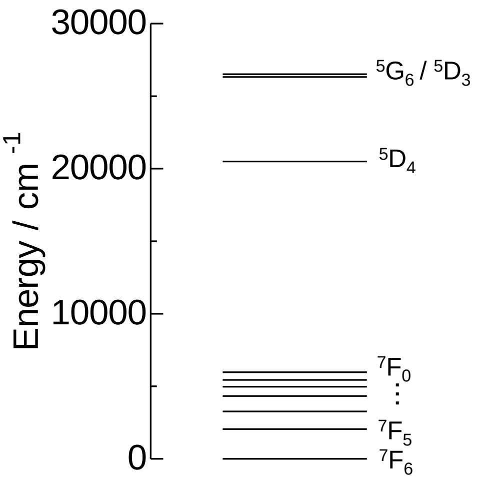

Figure S1. Energy diagram of Tb(III).

Supplementary Note 2. Emission quantum yield for Tb(III) complexes

Trivalent lanthanide ions show weak absorption (molar absorption coefficient (ε) of 4f-4f transitions < 10 M^{-1} cm^{-1}). This limitation can be overcome by using organic compounds that exhibit high light-absorption (ε = 10^3 to 10^5 M^{-1} cm^{-1}) as ligands in Ln^{III} complexes. Photosensitized emission quantum yield can be expressed using the following equation:

$$\phi_{\mathrm{tot}} = \eta_{\mathrm{sens}} \times \phi_{\mathrm{ff}} = \eta_{\mathrm{sens}} \times \frac{k_{\mathrm{r}}}{k_{\mathrm{r}}+k_{\mathrm{nr}}} \quad \text{(eq. S1)}$$

Here, Φ_{tot} and Φ_{ff} are the photosensitized (ligand-excited) and 4f-4f excited emission quantum yield, respectively, while η_{sens}, k_{r}, and k_{nr} are the efficiency of sensitization, radiative rate constant in 4f-4f transitions, and non-radiative rate constant in 4f-4f transitions, respectively. Among the lanthanide complexes, Tb(III) complexes show a relatively large energy gap (14,800 cm^{-1}) between emitting level ($^5D_4$) and highest accepting state in emission ($^7F_0$), resulting in a low non-radiative rate constant and a highly 4f-4f excited emission quantum yield (Φ_{ff}) in the lanthanide complexes.[S4] Thus, the photosensitized energy transfer efficiency is often a critical factor for high photosensitized emission quantum yield. The 4f-4f forbidden transition provides long-lived 4f-4f excited state (sub-millisecond ～millisecond order). Latva gave the "empirical law" for strong Tb(III) emission with long-lived excited states, in the lanthanide photochemistry history.[S5] They suggested a threshold for the energy level difference between energy of the T_1 level and the emitting level of Tb(III) ions ($^5D_4$: 20,500 cm^{-1}) for suppression of photon loss from back energy transfer (required energy gap between donor and acceptor in case of Tb(III) complexes > 1850 cm^{-1}). On the other hand, oxygen-dependent 4f-4f emission lifetime measurements revealed the formation of excited state equilibrium between the emitting level of Tb(III) ion and ligand T_1 state in some Tb(III) complexes[S6-S8]. Therefore, the rate of energy transfer between these two states was faster than the rate of deactivation from $^5D_4$ and T_1 toward the ground state. This shows that controlling the lifetime of T_1 phosphorescence is important for the efficient transfer of photosensitized energy.

[X-ray crystal data]

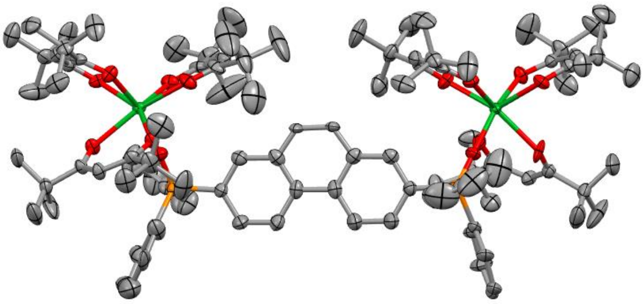

Figure S2. ORTEP drawings (ellipsoids set at 50% probability) of $[Lu_2(tmh)_6(dpph)]$.

Table S1. Crystallographic data for $[Tb_2(tmh)_6(dpph)]$ and $[Lu_2(tmh)_6(dpph)]$.

	$[Tb_2(tmh)_6(dpph)]$	$[Lu_2(tmh)_6(dpph)]$
chemical formula	$C_{104}H_{142}O_{14}P_2Tb_2$	$C_{104}H_{142}Lu_2O_{14}P_2$
crystal system	Triclinic	Triclinic
space group	P-1	P-1
a / Å	20.6292(3)	20.6743(3)
b / Å	21.8080(3)	21.7193(3)
c / Å	23.8231(3)	23.5751(3)
volume / $Å^3$	10599.8(3)	10467.7(3)
Z	2	4
density / g cm^{-3}	1.251	1.287
temperature / °C	–150	–150
R	0.0609	0.0814
wR_2	0.1696	0.2171

Supplementary Note 3. TD-DFT calculations for [Tb_2(tmh)$_6$(dpph)]

To analyze the S_0-T_1 transition characteristics, we performed TD-DFT calculation using the **Lu-dpph** structure obtained by single X-ray crystal analysis. The MWB60 basis set was adopted for Lu atoms[S9-S10], whereas the 6-31G(D) basis set was used for the other atoms. The S_0-T_1 transition is mainly composed of two electronic configurations (Figure S3), which is corresponding to the π-π* transition. The T_1 level was 19,220 cm^{-1}, which is similar to the experimentally estimation value (19,850 cm^{-1}). On the other hand, the S_0-T_2 transition is assigned to charge transfer transition from the tmh to dpph ligand. The T_2 level was 22,370 cm^{-1}, indicating a relatively large energy gap between the T_1 and T_2 state (ΔE(T_1-T_2) = 3,150 cm^{-1}). To further confirm the transition from the lowest excited state to ground state for **Lu-dpph**, the phosphorescence spectrum of the free dpph ligand was evaluated (Figure S4). The emission spectral shape and transition orbital characteristics evaluated by TD-DFT calculation (Figure S5) of the free dpph ligand are similar with those of **Lu-dpph**. Thus, the observed phosphorescence bands of **Lu-dpph** (Figure 3a) are mainly due to the localized π-π* transition character of the dpph moiety.

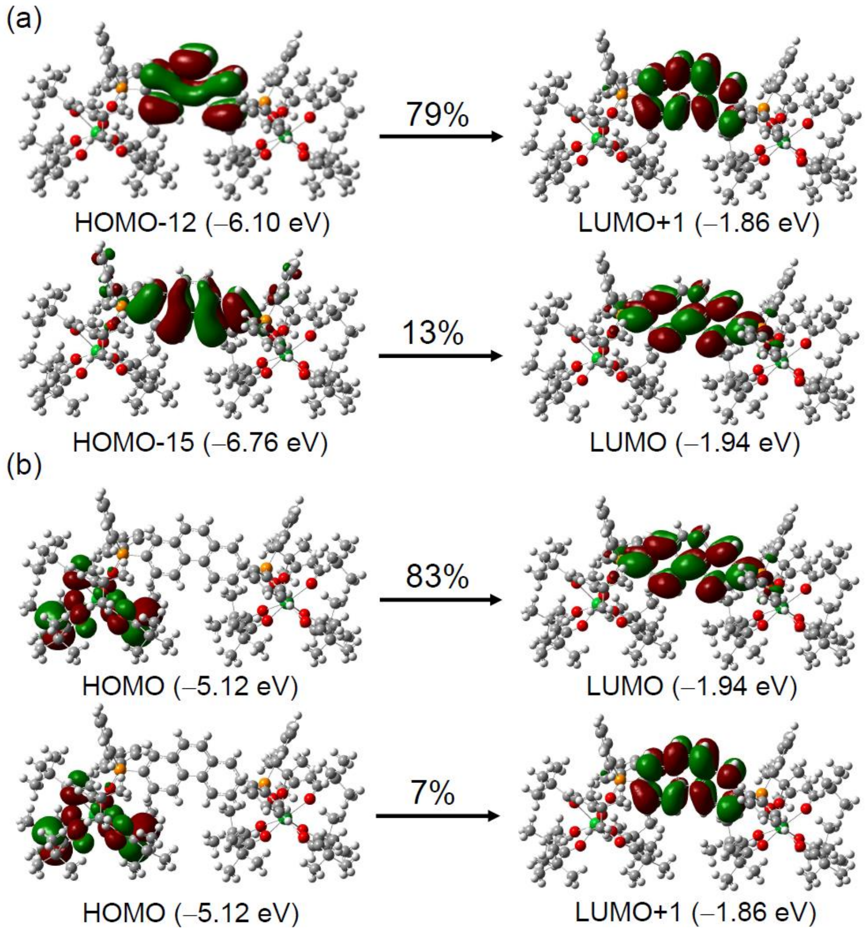

Figure S3. Main electronic configurations of (a) S_0-T_1 and (b) S_0-T_2 transitions for **Lu-dpph**.

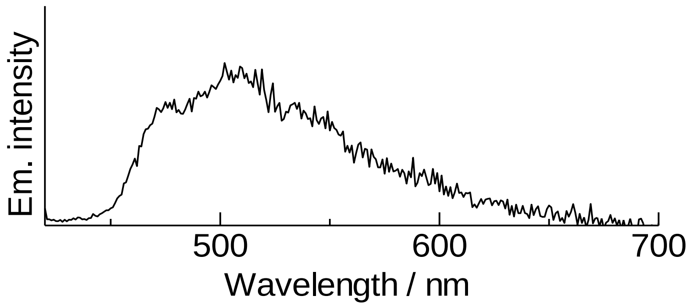

Figure S4. Phosphorescence spectrum of the dpph ligand, which were doped in β-estradiol (7 wt.%), at 293 K (delay time : 50 ms, λ_{ex} = 362 nm).

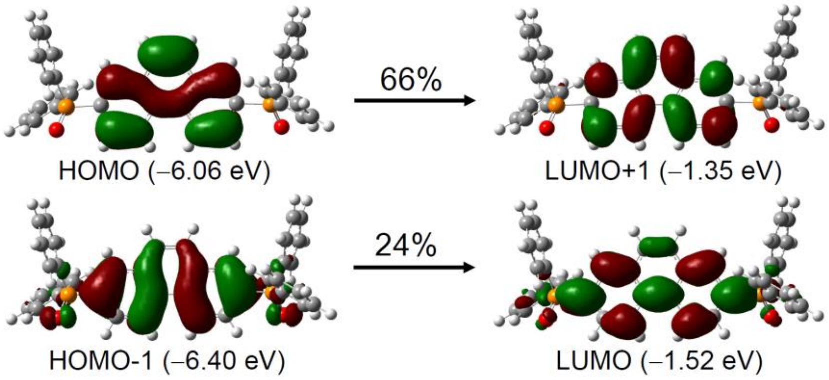

Figure S5 Main electronic configurations of S_0-T_1 transitions for the dpph ligand. Un-stabilized dpph LUMO (−1.94 eV → −1.52 eV) level by de-complexation, which are consistent with the blue-shifted phosphorescence band by de-complexation (Figure 3a and Figure S4).

Supplementary Note 4. Emission properties of Lu-dpph as a crystal

The emission decay (Ln-Ln plot) of **Lu-dpph** is shown in Figure S6. The decay shape was similar to sum up the exponential decay and power-law decay, which might suggest the existence of intermediate states for phosphorescence.[S11] The normalized emission spectra of **Lu-dpph** with/without delay time are shown in Figure S7. From the comparison of the two emission spectra (Figure S7, black line and red line), the emission spectra are expected to originate from the fluorescence band edge (Figure S11) and phosphorescence spectrum (Figure 3a) of the dpph moiety. The phosphorescence quantum yield of **Lu-dpph** under Ar condition (λ_{ex} = 400 nm) was estimated to be 1.3 %. The emission spectra for various delay times (10, 20, 30, 50, and 100 ms) are also shown in Figure S8. The emission spectral band (delay: 10 ms) was slightly red-shifted after increasing delay times (20, 30, 50, and 100 ms), which was attributed to the contribution of the dpph fluorescence band edge.

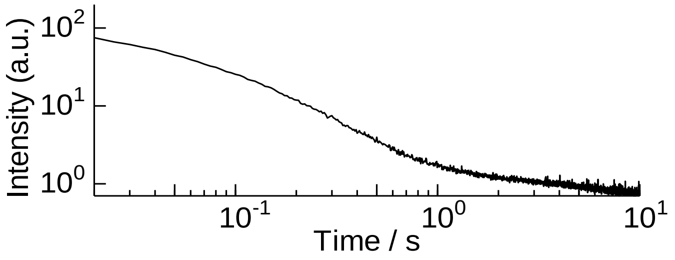

Figure S6. The emission decay curves (λ_{ex} = 400 nm, λ_{em} = 530 nm) of **Lu-dpph** at 293 K.

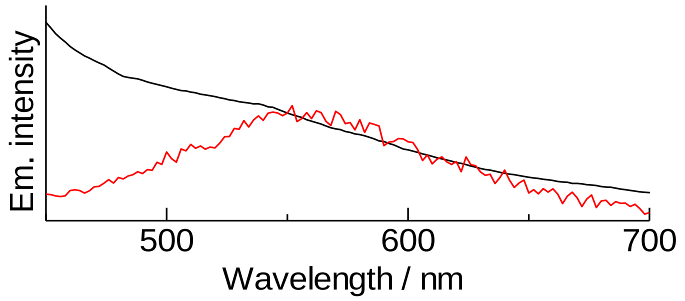

Figure S7. The emission spectra of **Lu-dpph** excited by 400 nm at 293 K (delay time, black line: 0 ms, red line: 20 ms). Normalized by emission intensity at 550 nm.

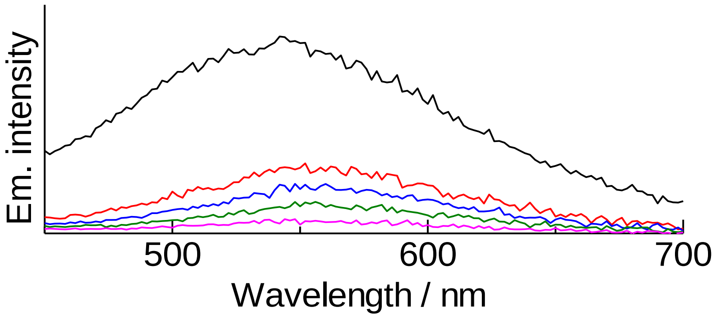

Figure S8. The emission spectra of **Lu-dpph** excited by 400 nm at 293 K (delay time, black line: 10 ms, red line: 20 ms, blue line: 30 ms, green line: 50 ms, and purple line: 100 ms) without normalization.

[Photophysical data]

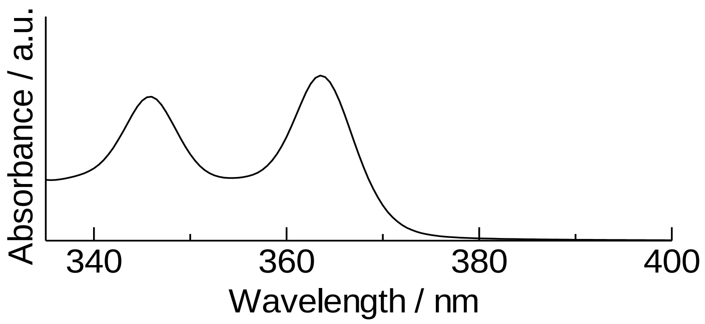

Figure S9. Electronic absorption spectrum of dpph ligand in chloroform (1.0×10^{-3} M).

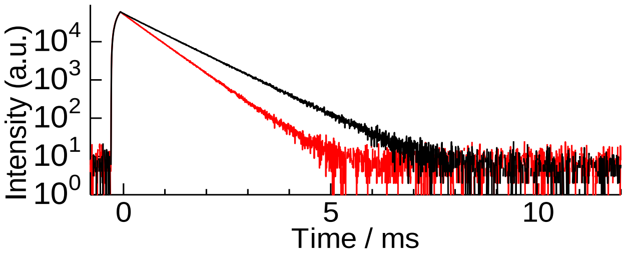

Figure S10. Emission decay curves of **Tb-dpph** in Ar (black line) and air (red line).

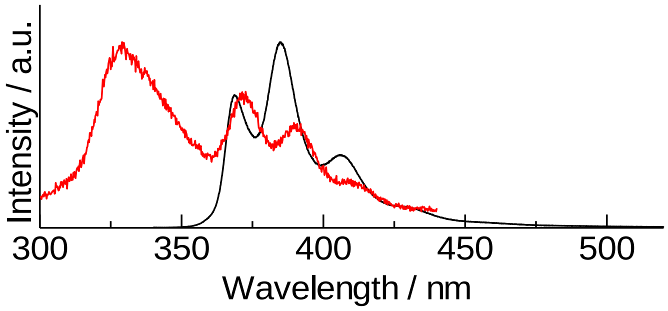

Figure S11. Emission spectra of dpph ligand (black line: chloroform, 1.0×10^{-3} M, λ_{ex}= 300 nm) and tmh ligands in $[Lu_2(tmh)_6]$ (red line: methanol, 1.0×10^{-4} M, λ_{ex}= 230 nm). The dpph ligand show the appropriate energy donating level (S_1 = 27,100 cm^{-1}) for $^5G_6$ (= 26,510 cm^{-1}) and $^5D_3$ (= 26,320 cm^{-1}) states of Tb(III) ion.

Supplementary Note 5

Temperature-dependent emission intensity for Tb-dpph

Temperature-dependent emission spectra (at 100, 150, 200, 250, 300, 350, and 400 K) excited by the dpph ligand (λ_{ex} =356 nm) were evaluated for **Tb-dpph** (Figure S12). The calculated emission area increased with an increase in temperature (Figure S13). The increased emission intensity with an increase in temperature (100–350 K) indicates the existence of thermally enhanced photosensitized emission pathways such as intersystem crossing[S12] and/or energy transfer from T_1. Non-increase in the emission intensity with increasing temperature (350–400 K) is considered to be originated from the increased non-radiative rate constant of $T_1 \rightarrow S_0$ transition in the dpph ligand.

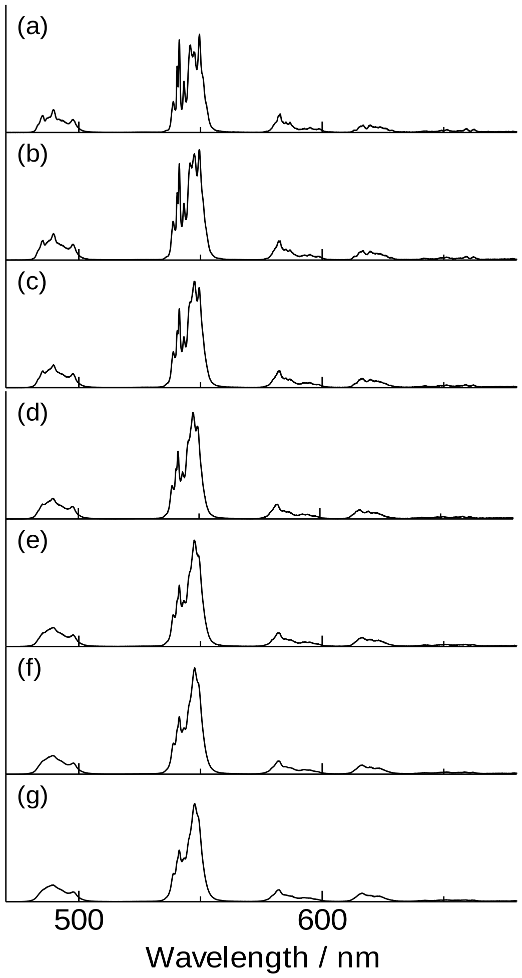

Figure S12. Temperature-dependent emission spectra (λ_{ex} = 356 nm, a: 100 K, b: 150 K, c: 200 K, d: 250 K, e: 300 K, f: 350 K, g: 400 K).

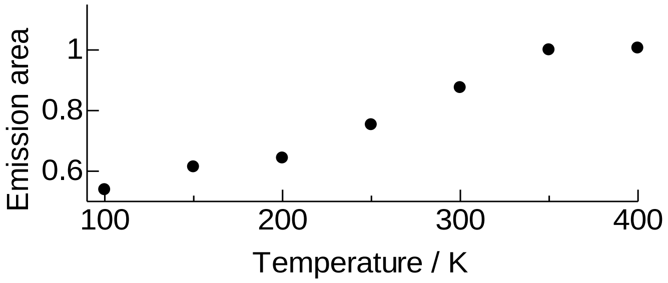

Figure S13. Temperature-dependent emission area (λ_{ex} = 356 nm). Normalized by maximum value at 350 K.

To clarify the excited state dynamics related to T_1 and $^5D_4$ levels, the emission properties by direct 4f-4f excitation of the Tb(III) ion (λ_{ex}= 482 nm) without the intersystem crossing pathway ($S_1 \rightarrow T_1$) were evaluated for **Tb-dpph**. The temperature-dependent emission spectra (Figure S14; at 100, 150, 200, 250, 300, 350, and 400 K) were evaluated for **Tb-dpph** using direct 4f-4f excitation (λ_{ex} = 482 nm). The calculated emission area increased with an increase in temperature in the range of 100–350 K and decreased with a further increase in temperature from 350 to 400 K (Figure S15). These photophysical data show the thermally enhanced emission intensity in the excited state equilibrium between the dpph triplet and $^5D_4$ (Tb), which is revealed by oxygen-dependent emission lifetime (Figure S10).[S5-S7] Thus, the photo-sensitized emission pathway contains the thermally enhanced energy transfer pathway corresponding to the $^7F_6 \rightarrow ^5D_4$ transitions.

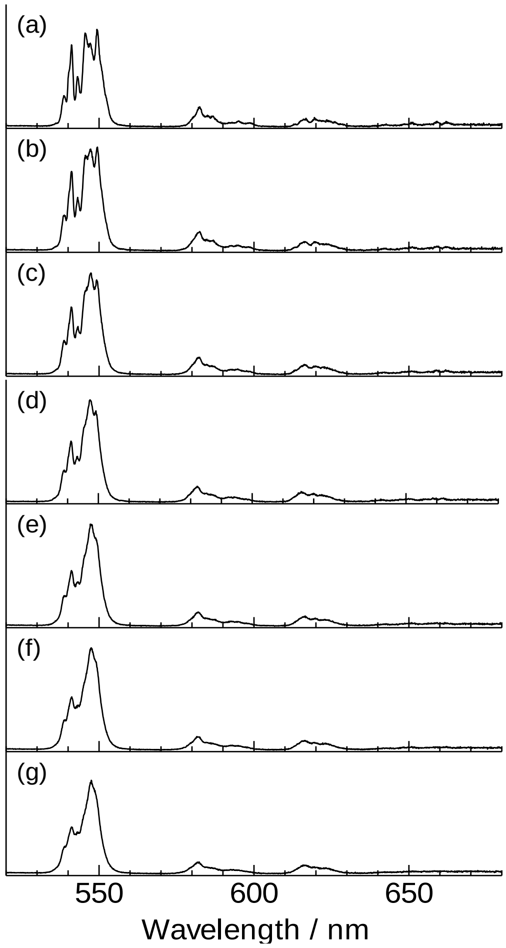

Figure S14. Temperature-dependent emission spectra (λ_{ex} = 482 nm, a: 100 K, b: 150 K, c: 200 K, d: 250 K, e: 300 K, f: 350 K, g: 400 K).

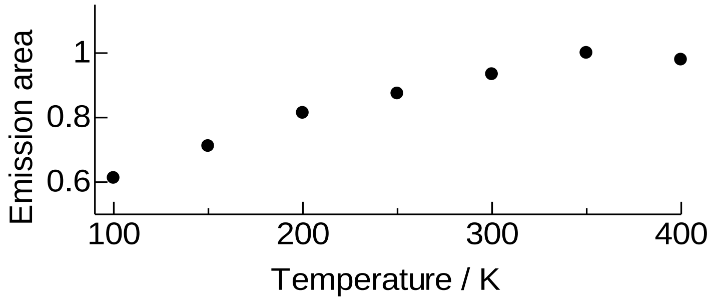

Figure S15. Temperature-dependent emission area (λ_{ex} = 482 nm). Normalized by maximum value at 350 K.

Supplementary Note 6

Temperature-dependent emission lifetimes for Tb-dpph and their comparison

We measured the temperature-dependent emission lifetimes using **Tb-dpph** (Figure S16-S17). The other data (Figure S18 $[Tb(tmh)_3(tppo)]$[S13] (tppo: triphenylphosphine oxide) and $[Tb_2(tmh)_6(bpeb)]$ (bpeb: 1,4-bis(diphenylphosphoryl)ethynyl)benzene)) are also shown in Figure S16 for comparison. The T_1 level of the extended π-conjugated bpeb ligand is estimated to be 21,230 cm^{-1}, from TD-DFT calculations (B3LYP/6-31G(D)) using the structure optimized by DFT calculations (B3LYP/6-31G(D)). The emission lifetimes of $[Tb(tmh)_3(tppo)]$ and $[Tb_2(tmh)_6(bpeb)]$, with the extended π-conjugated systems, are insensitive and sensitive to temperature, respectively. The temperature-dependent emission lifetime of $[Tb_2(tmh)_6(bpeb)]$ can be attributed to the deactivation pathway induced by back energy transfer from $^5D_4$ to T_1.[S5] In contrast, **Tb-dpph** has a large π-conjugated phenanthrene framework with a low T_1 level (19,850 cm^{-1}), and its emission lifetime is insensitive to temperature in the thermally-enhanced emission region (100 K – 350 K). In the excited dynamics system, an excited-state equilibrium between the T_1 and $^5D_4$ levels was revealed by the oxygen-dependent emission lifetime. These results indicate the existence of a mechanism for suppression of the quenching effect *via* the T_1 state with an excited-state equilibrium.

The equation of emission lifetime (τ_{obs}) in the excited equilibrium between donor and acceptor is generally expressed as follows.[S14-S15]

$$\frac{1}{\tau_{obs}} = \alpha\frac{1}{\tau_{donor}} + (1-\alpha)\frac{1}{\tau_{acceptor}} \quad \text{(eq. S2)}$$

$$K_{eq} = \frac{\alpha}{1-\alpha} = \frac{k_{A\to D}}{k_{D\to A}} \quad \text{(eq. S3)}$$

Herein, $k_{A\to D}$ and $k_{D\to A}$ are the energy transfer from acceptor (emission center) to donor and energy transfer from donor to acceptor, respectively. τ_{donor} and $\tau_{acceptor}$ correspond to the time constants for the decays of the excited donor and acceptor moieties, respectively. K_{eq} is the excited-state equilibrium constant. From the equation, an excited-state equilibrium, with an energy-donating state having a lower energy level than the energy-accepting state and a significantly lower deactivation rate to the ground state than that from the accepting state to the ground state, should provide a significantly longer lifetime; however, the emission lifetime of **Tb-dpph** is only slightly longer than that of other Tb(III) complexes containing tmh and phosphine oxide ligands (Figure S16). From the eq. S2, a larger value of $k_{D\to A}$ than that of $k_{A\to D}$ is a required condition for the temperature-insensitive emission lifetime in the excited state equilibrium; therefore, the simple endothermic energy transfer model is not appropriate to explain the **Tb-dpph** excited state dynamics.

The results suggest unusually efficient exothermic energy transfer pathways corresponding to the $^7F_5\to{}^5D_4$ transitions from the T_1 states ($+\Delta E$ = 1,400 cm^{-1}) besides the endothermic energy transfer pathways corresponding to the $^7F_6\to{}^5D_4$ transitions from the T_1 states ($-\Delta E$ = 650 cm^{-1}) (Figure 4b;

the energy levels have been summarized in Table S2). The $^7F_5$ level is significantly populated owing to a long decay lifetime of $^7F_5 \rightarrow ^7F_6$ in a relatively large energy gap between them (2,050 cm^{-1}),[S16–S19] allowing energy transfer from the $^7F_5$ level.[S20] Theoretical calculations also suggest a larger matrix element for the energy-transfer rate of the $^7F_5 \rightarrow ^5D_4$ transition than that of the $^7F_6 \rightarrow ^5D_4$ transition.[S21] Thus, the exothermic energy transfer corresponding to the $^7F_5 \rightarrow ^5D_4$ transition from the T_1 states is potential pathways for the construction of the temperature-insensitive 4f-4f emission lifetime property. The other possibility is the effective energy transfer pathway from the T_2 state induced by thermally activated reverse internal conversion[S22] from T_1. Theoretical calculations suggest that the T_2 level (22,370 cm^{-1}) in the ground state structure is close to the emitting level ($^5D_4$: 20,500 cm^{-1}) from $^7F_6$. These data suggest the possibility of equilibration between three or four electronic states (T_1(-$^7F_6$), T_1(-$^7F_5$) (or/and T_2(-$^7F_6$)), and $^5D_4$(-S_0)) by thermal stress. A fast energy-donating process *via* the T_1(-$^7F_6$) state is required to explain the temperature-insensitive emission lifetime. Although determining the exact photosensitization pathway is difficult, this is, to the best of our knowledge, the first example of efficient photosensitized emission *via* the T_1 state in a lanthanide complex with an organic ligand T_1 level lower than the emitting level of the Ln(III) ion.

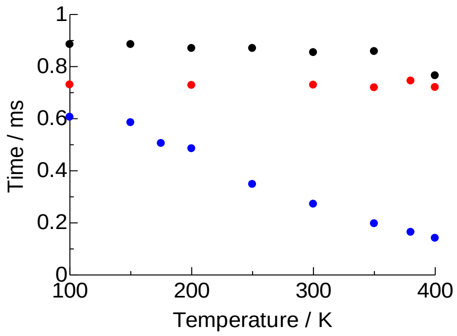

Figure S16. Temperature-dependent emission lifetimes (λ_{ex} = 355 nm, λ_{em} = 540 nm) for **Tb-dpph** (black dot), [Tb(tmh)$_3$(tppo)] (red dot)[S13], and [Tb$_2$(tmh)$_6$(bpeb)] (blue dot).

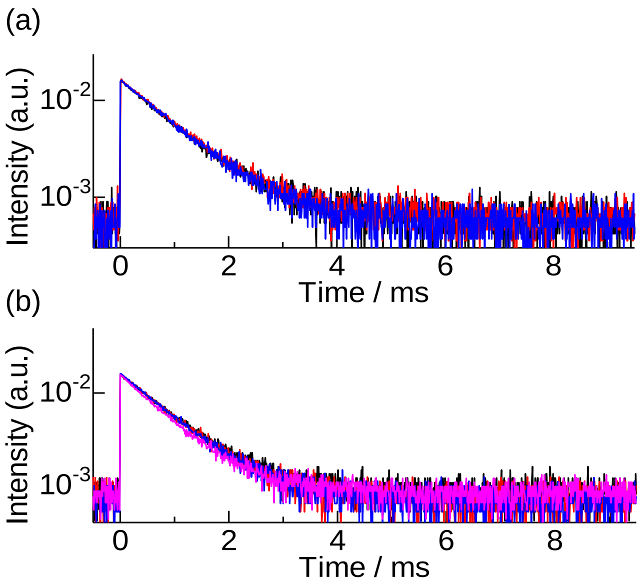

Figure S17. Temperature-dependent emission decays for **Tb-dpph** (λ_{ex} = 355nm, λ_{em} = 540 nm) (a, black line: 100 K, red line: 150 K, blue line: 200K) (b, black line: 250 K, red line: 300 K, blue line: 350 K, pink line: 400K).

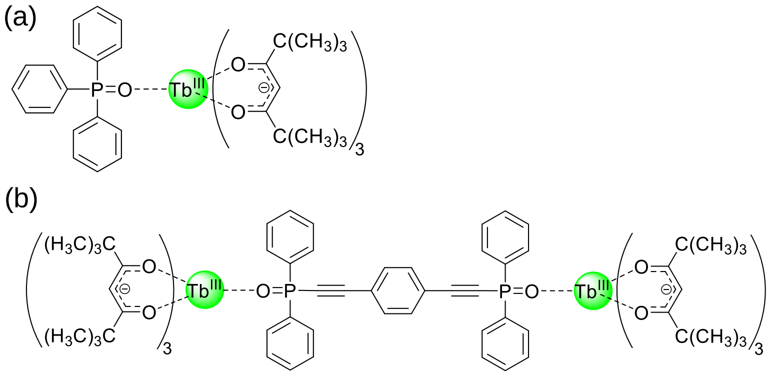

Figure S18. Chemical structures of (a) [Tb(tmh)$_3$(tppo)] and (b) [Tb$_2$(tmh)$_6$(bpep)].

Table S2. Energy levels of ligands and Tb(III).

	Energy / cm^{-1}
S_1 energy (dpph)	27,100
T_1 energy (dpph)	19,850
S_1 energy (tmh)	30,400
T_1 energy (tmh)	24,400
$^5G_6$ (Tb)	26,510 (24,460)[a]
$^5D_3$ (Tb)	26,320 (24,270)[b]
$^5D_4$ (Tb)	20,500 (18,450)[c]

a: Energy gap between $^5G_6$ and $^7F_5$. b: Energy gap between $^5D_3$ and $^7F_5$. c: Energy gap between $^5D_4$ and $^7F_5$.

Synthesis of [Tb_2(tmh)$_6$(bpep)] (Figure S18b)

[Tb_2(tmh)$_6$] (0.87 g, 0.6 mmol), and phosphine oxide ligand (bpep[S23]: 0.32 g, 0.6 mmol) were dissolved in methanol. The solution was refluxed for 12 h. The solution was concentrated by an evaporator, and then filtrated with addition of small amounts of methanol into the solution. That mixture was left at room temperature to recrystallize giving colorless crystals (Yield: 69%, 0.83 g, 0.41 mmol).

Elemental analysis calcd.(%) for $C_{100}H_{138}O_{14}P_2Tb_2$: C, 61.79; H, 7.16. Found C, 61.70; H, 7.16.

Supplementary Note 7

In order to discuss the enhancing effect of endothermic T_1-to-$^5D_4$ energy transfer on the emission intensity with increasing temperature, we fit the equation of the extent of enhancement on the quantum yield with increasing temperature $\Phi_{\text{tot}}(T) - \Phi_{\text{tot}}(100\text{K})$ to the actual data using the simplified model based on three excited levels (S_1, T_1 and $^5D_4$). In the given system in which back energy transfer occurs (diagram shown in Figure 4), the quantum yield can be expressed by the following equation.[S24]

$$\Phi_{\text{tot}} = \frac{A_{\text{r}}\{\eta_{\text{ISC}} W_{\text{FET,T}_1} + \eta_{\text{FET,S}_1}(A_{\text{T}_1} + W_{\text{FET,T}_1})\}}{(A_{\text{T}_1} + W_{\text{FET,T}_1})(A_{\text{r}} + A_{\text{nr}} + W_{\text{BET}}) - W_{\text{BET}} W_{\text{FET,T}_1}} \quad \text{(eq. S4)}$$

A_{r} and A_{nr} are the total radiative and the internal conversion rate constants of the $^5D_4$ state, respectively. η_{ISC} and $\eta_{\text{FET,S}_1}$ are the intersystem crossing and S_1-to-$^5D_4$ energy transfer efficiency, respectively. A_{T_1} is the total relaxation rate constant of the T_1 state. $W_{\text{FET,T}_1}$ and W_{BET} are the rate constants of T_1-to$^5D_4$ energy transfer and its reverse process, respectively. The $W_{\text{FET,T}_1}$ can be expressed by the Marcus-like equation:

$$W_{\text{FET,T}_1} = \frac{2\pi}{\hbar}\left|\langle S_0, {}^5D_4|V|T_1, {}^7F_6\rangle\right|^2 (4\pi\lambda k_{\text{B}}T)^{-\frac{1}{2}} \exp\left\{-\frac{\left(\lambda + \Delta G_{T_1 \to {}^5D_4}\right)^2}{4\lambda k_{\text{B}}T}\right\} \quad \text{(eq. S5)}$$

where, $\hbar$, k_{B}, and T is the Planck constant, Boltzmann constant, and temperature, respectively. $\langle T_1, {}^7F_6|V|S_0, {}^5D_4\rangle$ is the matrix element of the interaction energy, λ is the reorganization energy, and $\Delta G_{{}^5D_4 \to T_1}$ is the energy gap between the T_1 and $^5D_4$ states. T_1-to$^5D_4$ energy transfer is endothermic in **Tb-dpph**, which means that $\Delta G_{{}^5D_4 \to T_1}$ is a positive value of 650 cm^{-1}. Since the interaction energy between the two states $\langle S_0, {}^5D_4|V|T_1, {}^7F_6\rangle$ are the same regardless of the direction of the energy transfer process between the states, if we assume that the potential energy surfaces at the relevant energy scale are similar in shape (same λ), the reverse process W_{BET} can be expressed as:

$$W_{\text{BET}} = \frac{2\pi}{\hbar}\left|\langle S_0, {}^5D_4|V|T_1, {}^7F_6\rangle\right|^2 (4\pi\lambda k_{\text{B}}T)^{-\frac{1}{2}} \exp\left\{-\frac{\left(\lambda - \Delta G_{{}^5D_4 \to T_1}\right)^2}{4\lambda k_{\text{B}}T}\right\} \quad \text{(eq. S6)}$$

It should be noted that the only difference between eq. S5 and eq. S6 is the sign on $\Delta G_{{}^5D_4 \to T_1}$ in the exponent.

Here, we make two assumptions:

- Total of intersystem crossing and direct energy transfer to the $^5D_4$ state from the S_1 state occurs at unity ($\eta_{\text{FET,S}_1} + \eta_{\text{ISC}} = 1$). This may be a drastic assumption but can be reasonably true considering that the total quantum yield of **Tb-dpph** reaches over 80%, implying that the total energy transfer efficiency from the ligand to the Tb^{3+} ion is very efficient.
- The total relaxation rate constant of the $^5D_4$ state is the inverse of the emission lifetime at 100 K ($A_{\text{r}} + A_{\text{nr}} = 1130\ \text{s}^{-1}$).

With these assumptions, equation of $\Phi_{\text{tot}}(T) - \Phi_{\text{tot}}(100\text{K})$ is sufficiently simplified where T and

$\Phi_{tot}(T) - \Phi_{tot}(100K)$ are the variables, $A_r + A_{nr}$ and $\Delta G_{^5D_4 \to T_1}$ are fixed, allowing η_{ISC}, A_{T_1}, A_r (with $A_{nr} = 1130 - A_r$), $|\langle S_0, {}^5D_4|V|T_1, {}^7F_6\rangle|^2$, and λ to be the fitting parameters. Figure S19 shows the experimental data of $\Phi_{tot}(T) - \Phi_{tot}(100K)$ (calculated based on the quantum yield at 300 K and the emission intensity provided in Figure S12) along with the fitted line using the equation. This resulted in the value shown in Table S3. All of the values obtained are reasonable for lanthanide complexes. This result suggests that **Tb-dpph** has a higher quantum yield at higher temperatures owing to thermally-assisted energy transfer. However, this simplified model cannot explain its temperature-insensitive emission lifetime.

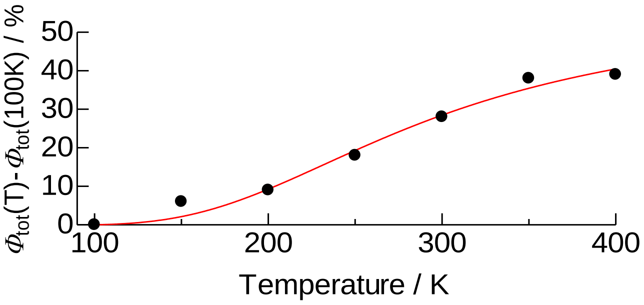

Figure S19. Experimental values of $\Phi_{tot}(T) - \Phi_{tot}(100K)$ (dots), and their fitted line (red) with the equation based on Figure 4 model system.

Table S3. Fit results of the temperature dependence of quantum yield.

Parameters	Values
η_{ISC}	0.366
A_{T1}	60.2 s^{-1}
A_r	712 s^{-1}
$|\langle S_0, {}^5D_4|V|T_1, {}^7F_6\rangle|^2$	0.0108 cm^{-2}
λ	1238 cm^{-1}
Reduced χ^2	1.28×10^{-3}
R^2	1.000
Adjusted R^2	1.000

Table S4. Fit results of the temperature dependence of quantum yield.

Parameters	100 K	150 K	200 K	250 K	300 K	350 K	400 K
W_{FET} / s^{-1}	3.9112 $\times10^2$	1.0089 $\times10^4$	4.9111 $\times10^4$	1.2377 $\times10^5$	2.2539 $\times10^5$	3.4173 $\times10^5$	4.6276 $\times10^5$
W_{BET} / s^{-1}	4.5066 $\times10^6$	5.1469 $\times10^6$	5.2717 $\times10^6$	5.2146 $\times10^6$	5.0907 $\times10^6$	4.9445 $\times10^6$	4.7945 $\times10^6$
Φ / %	45	51	54	63	73	83	84

Supplementary Note 8

Photophysical properties of Lu-dpph and Tb-dpph dispersed in β-estradiol

We also investigated the photophysical properties of **Lu-dpph** and **Tb-dpph** doped in solid host. Herein, β-estradiol was selected as a rigid amorphous host with a relatively high T_1 levels (22,580 cm^{-1}).[S25] **Lu-dpph** and **Tb-dpph** were doped in β-estradiol (20 and 3 wt.%, respectively) at 185 °C and the samples were cooled to room temperature. The emission spectrum and emission decay behavior of **Lu-dpph** in β-estradiol (Figure S20) was similar to those of **Lu-dpph** in the crystal state. The emission spectrum and emission lifetime (τ = 0.84 ms) of **Tb-dpph** in β-estradiol (Figure S21) was also similar to those of **Tb-dpph** in crystal state. The excitation spectra exhibit a strong band at approximately 360 nm, indicating effective photosensitization from the dpph ligand. These results suggest that effective intermolecular interactions between Tb(III) complexes are not necessarily required for thermally assisted energy transfer from the lower T_1 level to the emitting level of Tb(III). Therefore, we believe that the combination of Tb(III) and organic ligands with a low T_1 level and transition probability ($T_1 \rightarrow S_0$) in a rigid amorphous host would be one of the novel strategies for the preparation of bright luminescent Tb(III) complexes.

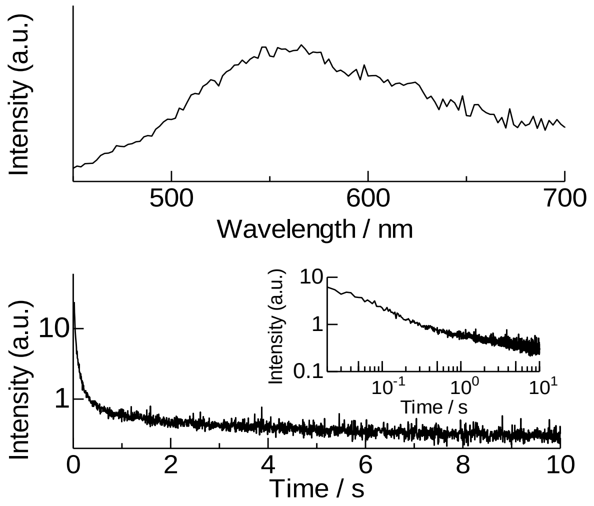

Figure S20. The emission spectrum (a: λ_{ex} = 370 nm; delay: 20 ms) and decay curves (b: λ_{ex} = 400 nm; λ_{em} = 530 nm) of **Lu-dpph** at 293 K.

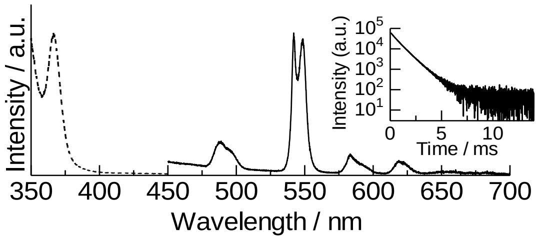

Figure S21. The emission (a: λ_{ex} = 356 nm, solid line) and excitation spectra (λ_{em} = 548 nm, broken line) of **Tb-dpph** at 293 K. Inset: Emission decay curve (b: λ_{ex} = 356 nm; λ_{em} = 548 nm) at 293 K.

Supplementary Note 9

Advantages of photosensitization with a low T_1 level for enhancement of brightness

Tb(III) complexes with high emission quantum yields (≧ 70%) have been already reported.[S4, S8, S26-S29] The focus of this study was achieving highly efficient photosensitization using a donating ligand with a low T_1 level, which resulted in the excitation of the low S_1 level, resulting in an effective low-energy-driven photosensitization.[S30] The energy-accepting level of the Tb(III) ion was relatively high ($^5D_4$: 20,500 cm^{-1}), requiring significantly high T_1 and S_1 level in the organic ligands for effective photosensitization. Brightness is defined by the product of molar absorption coefficient and emission quantum yield.[S31] For example, [Tb(tmh)$_3$(tppo)] also produces a highly emission quantum yield (Φ = 66 %)[S13] but its brightness, when excited using UV-light (λ = 365 nm), is estimated to be approximately 6 M^{-1} cm^{-1}, based on the molar absorption coefficient (Figure S22a, ε_{365nm} = 9 M^{-1} cm^{-1}). In contrast, the brightness of **Tb-dpph** excited using UV-light (λ = 365 nm) was estimated to be 1,300 M^{-1} cm^{-1} (Figure S22b, ε_{365nm} = 1,780 M^{-1} cm^{-1}). Thus, our concept is expected to be useful for improving light-absorption properties and brightness.

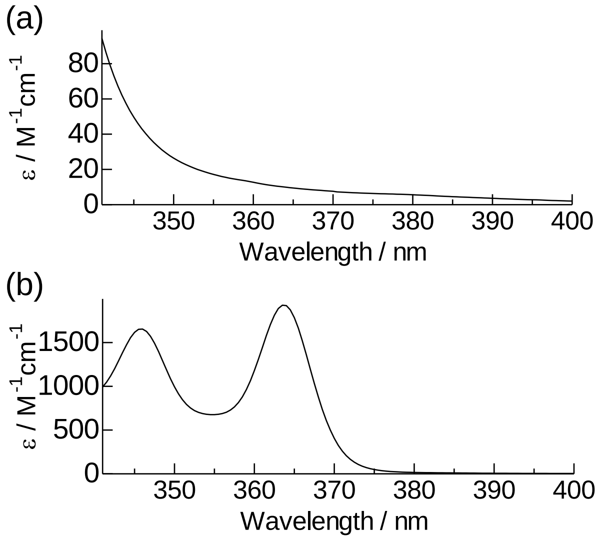

Figure S22. The electronic absorption spectra of [Tb(tmh)$_3$(tppo)] in toluene (a, 5.0 × 10^{-3} M) and **Tb-dpph** in chloroform (b, 5.6 × 10^{-4} M).

Supplementary References

S1. D. Y. Medina-Velazquez, U. Caldiño, A. Morales-Ramirez, J. Reyes-Miranda, R. E. Lopez, R. Escudero, R. Ruiz-Guerrero, M. F. Morales, Synthesis of luminescent terbium-thenoyltriflouroacetone MOF nanorods for green laser application. *Opt. Mater.* **87**, 3 (2019).

S2. K. S. Thomas, S. Singh, and G. H. Dieke, Energy levels of Tb in $LaCl_3$ and other chlorides, *J. Chem. Phys.* **38**, 2180 (1963).

S3. P. Dorenbos, The $4f^n \leftrightarrow 4f^{n-1}5d$ transitions of the trivalent lanthanides in halogenides and chalcogenides, *J. Lumin.* **91**, 91 (2000).

S4. Bünzli, J.-C. G. On the design of highly luminescent lanthanide complexes. *Coord. Chem. Rev.* **293–294**, 19 (2015).

S5. Latva, M., Takalo, H., Mukkala, V.-M., Matachescu, C., Rodríguez-Ubis, J. C., Kankare, J. Correlation between the lowest triplet state energy level of the ligand and lanthanide(III) luminescence quantum yield. *J. Lumin.* **75**, 149 (1997).

S6. Sørensen, T. J., Kenwright, A. M., Faulkner, S. Bimetallic lanthanide complexes that display a ratiometric response to oxygen concentrations. *Chem. Sci.* **6**, 2054 (2015).

S7. Sabbatini, N., Guardigli, M., Manet, I., Bolletta, F., Ziessel, R. Synthesis and luminescence of lanthanide complexes of a branched macrocyclic ligand containing 2,2'-bipyridine and 9-methyl-1,10-phenanthroline subunits. *Inorg. Chem.* **33**, 955 (1994).

S8. Kitagawa, Y. et al., Effective photosensitization in excited-state equilibrium: Brilliant luminescence of Tb^{III} coordination polymers through ancillary ligand modifications. *ChemPlusChem*, e202200151 (2022).

S9. M. Dolg, H. Stoll, and H. Preuss, A combination of quasirelativistic pseudopotential and ligand field calculations for lanthanoid compounds, *Theor. Chim. Acta* **85**, 441 (1993).

S10. M. Dolg, H. Stoll, A. Savin, and H. Preuss, Energy-adjusted pseudopotentials for the rare earth elements, *Theor. Chim. Acta* **75**, 173 (1989).

S11. Lin, Z., Kabe, R., Wang, K., Adachi, C. Influence of energy gap between charge-transfer and locally excited states on organic long persistence luminescence. *Nat. Commun.* **11**, 191 (2020).

S12. Widman, R. P., Huber, J. R. Temperature effects in the intersystem crossing process of anthracene. *J. Phys. Chem.* **76**, 1524 (1972).

S13. Ferreira da Rosa, P. P. et al. Thermosensitive seven-coordinate Tb^{III} complexes with LLCT transitions. *Eur. J. Inorg. Chem.* **2018**, 2031 (2018).

S14. Denisov, S. A., Cudré, Y., Verwilst, P., Jonusauskas, G., Marín-Suárez, M., Fernández-Sánchez, J. F., Baranoff, E., McClenaghan, N. D. Direct Observation of Reversible Electronic Energy Transfer Involving an Iridium Center. *Inorg. Chem.* **53**, 2677 (2014).

S15. McClenaghan, N. D., Leydet, Y., Maubert, B., Indelli, M. T., Campagna, S. Excited-state equilibration: A process leading to long-lived metal-to-ligand charge transfer luminescence in

supramolecular systems. *Coord. Chem. Rev.* **249**, 1336 (2005).
S16. Rademaker, K. et al, Optical properties of Nd^{3+} and Tb^{3+}-doped KPb_2Br_5 and $RbPb_2Br_5$ with low nonradiative decay. *J. Opt. Soc. Am. B* **21**, 2117 (2004).
S17. Roy, U. N. et al., Tb^{3+}-doped KPb_2Br_5: Low-energy phonon mid-infrared laser crystal. *Appl. Phys. Lett.* **86**, 151911 (2005).
S18. Seven-coordinated lanthanide complexes with tmh and phosphine oxide ligands have provided a relatively small non-radiative rate constant in the emission process.[S20] These types of structures might be key for construction of long-lived $^7F_5$ in Tb^{III} complexes with vibrational organic ligands.
S19. Yanagisawa, K. et al., Seven-coordinate luminophores: Brilliant luminescence of lanthanide complexes with C_{3v} geometrical structures. *Eur. J. Inorg. Chem.* **2015**, 4769 (2015).
S20. Carneiro Neto, A. N. et al., On the long decay time of the $^7F_5$ level of Tb^{3+}. *J. Lumin.* **248**, 118933 (2022).
S21. Moura, R. T., Theoretical evidence of the singlet predominance in the intramolecular energy transfer in ruhemann's purple Tb(III) complexes. *Adv. Theory Simul.* 4, 2000304 (2021)
S22. H. F. Higginbotham et al., Heavy-Atom-Free Room-Temperature Phosphorescent organic light-emitting diodes enabled by excited states engineering. *ACS Appl. Mater. Interfaces* **13**, 2899 (2021).
S23. Hirai, Y. et al., Luminescent coordination glass: Remarkable morphological strategy for assembled Eu(III) complexes. *Inorg. Chem.* **54**, 4364 (2015).
S24. Omagari, S. et al. Toward accurate measurement of the intrinsic quantum yield of lanthanide complexes with back energy transfer, *Phys. Chem. Chem. Phys.* **22**, 3683 (2020).
S25. Hirata, S. et al. Efficient persistent room temperature phosphorescence in organic amorphous materials under ambient conditions. *Adv. Funct. Mater.* **23**, 3386 (2013).
S26. Correia, S. F. H. et al, High emission quantum yield Tb^{3+}-activated organic-inorganic hybrids for UV-down-shifting green light-emitting diodes. *Eur. J. Inorg. Chem.* **2020**, 1736 (2020).
S27. Chen, B.-L. et al., A thermostable terbium(III) complex with high fluorescence quantum yields. *New J. Chem.* **46**, 11021 (2022).
S28. Xia, T. et al., A terbium metal–organic framework for highly selective and sensitive luminescence sensing of Hg^{2+} ions in aqueous solution. *Chem.–Eur. J.* **22**, 18429 (2016).
S29. Aquino, L. E. N. et al., Seven-coordinate Tb^{3+} complexes with 90% quantum yields: high-performance examples of combined singlet- and triplet-to-Tb^{3+} energy-transfer pathways. *Inorg. Chem.* **60**, 892 (2021).
S30. Kitagawa, Y. et al., Stacked nanocarbon photosensitizer for efficient blue light excited Eu(III) emission. *Commun. Chem.* **3**, 3 (2020).
S31. Wong, K.-L., Bünzli, J.-C. G., Tanner, P. A. Quantum yield and brightness. *J. Lumin.* **224**, 117256 (2020).
