# Supplementary material for: Thermally-assisted photosensitized emission in a trivalent terbium complex
Source: Commun Chem. 2023 Jun 22;6:122. doi: 10.1038/s42004-023-00922-5 (PMC10287744; doi:10.1038/s42004-023-00922-5)
Supplement: Supplementary file 3 — Description of additional supplementary files [file 42004_2023_922_MOESM3_ESM.pdf]

Description of Additional Supplementary Files

File Name: Supplementary Data 1

Description: Crystallographic data in CIF format for Tb(III) complex

File Name: Supplementary Data 2

Description: Crystallographic data in CIF format for Lu(III) complex
